# Extracellular Citrate Fuels Cancer Cell Metabolism and Growth

**DOI:** 10.3389/fcell.2020.602476

**Published:** 2020-12-04

**Authors:** Sebastian Haferkamp, Konstantin Drexler, Marianne Federlin, Hans J. Schlitt, Mark Berneburg, Jerzy Adamski, Andreas Gaumann, Edward K. Geissler, Vadivel Ganapathy, E. Kenneth Parkinson, Maria E. Mycielska

**Affiliations:** ^1^Department of Dermatology, University Medical Center, Regensburg, Germany; ^2^Department of Conservative Dentistry and Periodontology, University Medical Center, Regensburg, Germany; ^3^Department of Surgery, University Medical Center Regensburg, Regensburg, Germany; ^4^Research Unit Molecular Endocrinology and Metabolism, Helmholtz Zentrum München, German Research Center for Environmental Health, Neuherberg, Germany; ^5^Lehrstuhl für Experimentelle Genetik, Technische Universität München, Munich, Germany; ^6^Department of Biochemistry, Yong Loo Lin School of Medicine, National University of Singapore, Singapore, Singapore; ^7^Institute of Pathology, Kaufbeuren-Ravensburg, Kaufbeuren, Germany; ^8^Department of Cell Biology and Biochemistry, Texas Tech University Health Sciences Center, Lubbock, TX, United States; ^9^Center for Immunobiology and Regenerative Medicine, Barts and The London School of Medicine and Dentistry, Blizard Institute, London, United Kingdom

**Keywords:** cancer, metabolism, transporter, cancer associated fibroblast (CAF), senescent fibroblasts

## Abstract

Cancer cells need excess energy and essential nutrients/metabolites not only to divide and proliferate but also to migrate and invade distant organs for metastasis. Fatty acid and cholesterol synthesis, considered a hallmark of cancer for anabolism and membrane biogenesis, requires citrate. We review here potential pathways in which citrate is synthesized and/or supplied to cancer cells and the impact of extracellular citrate on cancer cell metabolism and growth. Cancer cells employ different mechanisms to support mitochondrial activity and citrate synthesis when some of the necessary substrates are missing in the extracellular space. We also discuss the different transport mechanisms available for the entry of extracellular citrate into cancer cells and how citrate as a master metabolite enhances ATP production and fuels anabolic pathways. The available literature suggests that cancer cells show an increased metabolic flexibility with which they tackle changing environmental conditions, a phenomenon crucial for cancer cell proliferation and metastasis.

## Introduction

To successfully grow and metastasise, cancer cells need to adjust their metabolism to fulfill high-energy requirements. The essential metabolites necessary for this process can be obtained from blood, synthesized *de novo*, or supplied by cancer-associated stromal cells. Uptake or release of different substrates from and to the extracellular space can occur through specific transporters expressed in the plasma membrane. The same holds true for the transport across the mitochondrial inner membrane where several transporters are present. Control of transporter expression is one of the ways by which metabolite uptake and release can be regulated. Differences in expression of the plasma membrane and mitochondrial transporters take part in the regulation of various metabolic pathways in the cytoplasm and mitochondria. Several transporters for specific nutrients and metabolites have been shown to be upregulated in cancer cells such as glucose, lactate or amino acids transporters (Ganapathy et al., 2009; Bhutia et al., 2015; El Ansari et al., 2018; Ma et al., 2018; Brown and Ganapathy, 2020; Brown et al., 2020b; Zhang and Li, 2020).

Synthesis of the building blocks necessary for the process of proliferation and membrane biogenesis requires increased production of fatty acids and cholesterol, considered a hallmark of cancer. Cytoplasmic citrate is the primary substrate in this process (Table 1). Until recently, it was believed that cancer cells cannot obtain citrate from the extracellular space and that the entire pool of cellular citrate arises from intracellular synthesis, mainly in the reverse Krebs cycle through reductive carboxylation from glutamine (Metallo et al., 2011), and to a much lesser extent, from glucose-derived pyruvate. Indeed, increased glutamine uptake has been associated with cancer growth (Bhutia and Ganapathy, 2016). However, some recent studies have shown that cancer cells are glutamine-dependent *in vitro*, whilst glutamine uptake rate is similar to normal cells when the same cancer cells are grown *in vivo* (Davidson et al., 2016). Moreover, radiotracer studies of glutamine uptake in mouse brain implanted with primary human tumors have shown that increased glutamine uptake occurs in the tissue surrounding the cancer but not in the cancer itself (Marin-Valencia et al., 2012). Therefore, the way citrate is synthesized in cancer cells might depend on the surrounding metabolic conditions.

**Table 1:** Reactions involved in the conversion of citrate into fatty acids and cholesterol in the cytoplasm.

**Reaction**	**Enzyme(s)**
Citrate + ATP + CoA → Acetyl-CoA + Oxaloacetate + ADP + Pi	ATP-Citrate lyase
Acetyl-CoA + CO_2_ + ATP → Malonyl-CoA + ADP + Pi	Acetyl-CoA carboxylase
Malonyl-CoA → → → Fatty acyl-CoA	Fatty acid synthase
Citrate + ATP + CoA → Acetyl-CoA + Oxaloacetate + ADP + Pi	ATP-Citrate lyase
3 Acetyl-CoA → HMG-CoA + 2 CoA	Thiolase and HMG-CoA synthase 1
HMG-CoA + 2 NADPH + 2 H^+^ → Mevalonate + CoA + 2 NADP^+^	HMG-CoA reductase
Mevalonate → → → Cholesterol	Several enzymes

We have recently discovered that cancer cells are able to take up citrate through the plasma membrane variant (pmCiC) of the mitochondrial (mCiC) citrate carrier (SLC25A1) belonging to the *SLC25* gene family (Mazurek et al., 2010). Our studies have also shown that uptake of extracellular citrate supports cancer metabolism, proliferation, fatty acid and protein synthesis (Mycielska et al., 2018). There is also another citrate transporter in the plasma membrane (SLC13A5) belonging to the *SLC13* gene family (Willmes et al., 2018; Jaramillo-Martinez et al., 2020). Here, we will discuss the transporters and the metabolic pathways that are involved in the uptake and utilization of extracellular citrate in cancer cells and the impact of extracellular citrate on cancer cell metabolism and growth.

## The Pathways Supporting Mitochondrial Activity in Cancer

Mitochondria consist of two membranes, an inner membrane that is impermeable to small molecules and an outer membrane that is permeable to small molecules. The impermeable nature of the inner membrane necessitates the presence of specific transporters to facilitate exchange of metabolites and nutrients between mitochondrial matrix and cytoplasm. As such, the inner membrane is rich in transporters, all of which belong to the SLC25 gene family; these transporters mediate the movement of a wide variety of metabolites in and out of the mitochondrial matrix (Gutiérrez-Aguilar and Baines, 2013). Abundance of these transporters in the mitochondrial membrane regulates the exchange rate of the substrates with cytoplasm and in consequence respective cytosolic and matrix metabolic pathways.

Fatty acid synthesis occurs in the cytoplasm, for which citrate is the primary substrate; this pathway is activated in cancer and it is a metabolic hallmark of cancer cells (Wang et al., 2015). Citrate is produced in the Krebs cycle within the mitochondrial matrix from the condensation of acetyl-CoA and oxaloacetate. In most cells, citrate is further metabolized into isocitrate through the action of mitochondrial aconitase (m-ACN/ACO2), which then goes through the rest of the reactions in the Krebs cycle. One notable exception is the prostate epithelial cell. The Krebs cycle is truncated in this cell type where citrate generated in the matrix fails to go through the next step mediated by m-ACN because of markedly reduced activity of this enzyme. This unique metabolic phenotype renders prostate epithelial cells net citrate producers. Citrate thus generated is then secreted into the prostatic fluid to facilitate the maturation and motility of spermatozoa. The reduced activity of m-ACN in these cells is due to Zn^2+^-mediated inhibition, and prostate epithelial cells exhibit a robust capacity to accumulate this metal. This allows the ratio of citrate to isocitrate to increase to 30–40:1 in these cells (Costello et al., 1976). As citrate fails to go through the Krebs cycle within the matrix in these cells, it accumulates and gets transported out of the matrix into the cytoplasm via the citrate transporter SLC25A1 in the inner membrane, which occurs in exchange for malate (i.e., citrate enters the cytoplasm and malate enters the matrix). Once in the cytoplasm, citrate is released into the luminal space via pmCiC, an alternative splice variant of SLC25A1 (Mycielska et al., 2009; Mazurek et al., 2010) and most likely also via the recently described citrate exporter ANKH (SLC62A1) (Szeri et al., 2020). The metabolic phenotype of the normal prostate epithelium as a citrate producer is reversed upon transformation into cancer cells. As a consequence, normal prostate has high concentrations of citrate whereas prostate cancer has low concentrations of citrate, a metabolic distinction that is exploited in the clinics for differential and minimally invasive diagnosis of prostate cancer (Banerjee et al., 2017; Braadland et al., 2017). This switch is facilitated by the loss of ability to accumulate Zn^2+^ during oncogenic transformation, which relieves m-ACN from the Zn^2+^-mediated inhibition (Costello and Franklin, 2006). The net outcome is the reduced levels of citrate inside the cancer cells. This ability of prostate cancer cells to oxidize citrate is surprising, as this process would lead to reduced net citrate accumulation whilst cancer cells need excess citrate level to support fatty acid synthesis in cytoplasm. How, therefore, do cancer cells manage to supply cytoplasm with the necessary amount of citrate?

**Figure 1:**
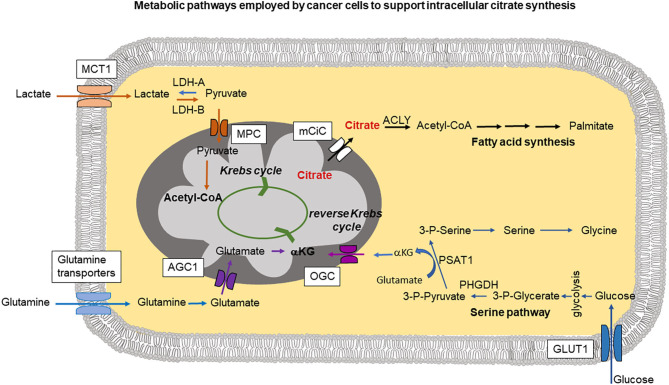
The cartoon summarizes intracellular pathways used by cancer cells to support increased citrate synthesis in mitochondria. This can occur through a serine pathway, where increased expression of phosphoglycerate dehydrogenase (PHGDH) creates a diversion from glycolysis. PSAT1 (phosphoserine aminotransferase) reaction requires conversion of glutamate into αKG (α-ketoglutarate) which can be then transported into mitochondria by oxoglutarate carrier (OGC) and support mitochondrial citrate synthesis through the reverse Krebs cycle. Lactate taken up through MCT1 (monocarboxylate transporter 1) is metabolized by lactate dehydrogenase-A (LDH-A) into pyruvate imported into mitochondria through MPC (mitochondrial pyruvate carrier) followed by Acetyl-CoA synthesis which can then join the Krebs cycle. Part of glutamine pool can be metabolized into glutamate and transported into mitochondria through AGC1 (aspartate-glutamate carrier isoform 1) followed by conversion into αKG. Released into the cytoplasm citrate is metabolized by ATP-citrate lyase (ACLY) and plays a central role in fatty acid synthesis.

It was widely believed that cancer cells did not take up citrate from the circulation (blood levels of citrate, ~200 μM) and that they met their increased demands for this metabolite via de no synthesis from glutamine (Metallo et al., 2011). This requires a novel reprogramming of the metabolism involving the reversal of the Krebs cycle in which α-ketoglutarate arising from glutamine gets converted into citrate by a process known as “reductive carboxylation.” Indeed, uptake of glutamine resulting from increased expression of multiple glutamine transporters has been associated with cancer cells (Bhutia and Ganapathy, 2016; Wang et al., 2020; Figure 1). Further studies indicated that glutamine addiction of cancer cells might depend on the metabolic environment. In particular, it has been determined that whilst there is a clear dependence on glutamine of human lung cancer cells *in vitro*, the same cells when injected in mice, synthesize citrate from glucose rather than glutamine (Davidson et al., 2016). Therefore, the metabolic environment of the cancer cells dictates the choice of extracellular metabolites and biochemical pathways used for satisfying the increased demands for citrate. A similar phenomenon of glutamine independence of cancer cells *in vivo* has been shown using primary cultures of human glioblastoma. In line with the previous report, when injected into mice, human glioblastoma cells did not show any increase in the uptake of glutamine. Interestingly, increased uptake of glutamine was observed in the stromal cells present in the tumor environment (Marin-Valencia et al., 2012). This increase in glutamine uptake would be consistent with cancer-associated stroma synthesizing metabolic substrates to support cancer cells; this could potentially include citrate.

Citrate synthesis in mitochondria from glutamine through a truncated (and reverse) Krebs cycle as postulated by several research groups (Metallo et al., 2011; Mullen et al., 2014) requires increased efforts to supply mitochondria with sufficient amounts of intermediates. Therefore, some additional pathways in cancer cells have been identified that could be used to fulfill the increased mitochondrial needs. A serine pathway has been recently postulated to be an alternative means to produce α-ketoglutarate (Locasale et al., 2011; Possemato et al., 2011). According to these studies, increased expression of phosphoglycerate dehydrogenase (PHGDH) siphons the glycolytic intermediate 3-phosphoglycerate for increased synthesis of serine and glycine (Figure 1). Serine and glycine are abundant in the plasma, and depletion of PHGDH could not decrease intracellular levels of these metabolites, suggesting that uptake of extracellular serine and glycine is also a likely contributor to these two amino acids in cancer cells. Suppression of PHGDH resulted, however, in decreased α-ketoglutarate levels which can be used to support Krebs cycle activity. This additional α -ketoglutarate synthesis could be potentially the main reason for cancer cells to use serine pathway (Possemato et al., 2011; Figure 1). In addition to the widely recognized role of serine and glycine in one-carbon metabolism, an obligatory pathway for any highly proliferating cell, including cancer cells, these amino acids also participate in the synthesis of α-ketoglutarate for use in the Krebs cycle in the forward direction to generate NADH and FADH_2_ for subsequent ATP production and also in the Krebs cycle in the reverse direction for reductive carboxylation for subsequent citrate production.

Another metabolite that has been implicated in support of cancer cell mitochondrial metabolism and in consequence in citrate synthesis, is lactate (Figure 1). Lactate can be imported into the cell through monocarboxylate transporter MCT1, heavily overexpressed in majority of aggressive tumors (Park et al., 2018; Tasdogan et al., 2020). Intracellularly, lactate can be converted into pyruvate and then into acetyl-CoA to feed into the Krebs cycle. Moreover, the conversion of lactate to pyruvate is accompanied by reduction of NAD^+^ into NADH, which suppresses the activity of glyceraldehyde-3-phosphate dehydrogenase in the direction of glucose breakdown but reverses the reaction to go in the opposite direction, thus sparing glucose 6 phosphate to feed into the pentose-phosphate pathway (Tasdogan et al., 2020). The pentose phosphate pathway is a major mechanism for production of NADPH, a reducing equivalent obligatory for anabolic pathways that are essential for cancer cells. Increased MCT1 expression and excess pentose-phosphate pathway activity have been shown to be necessary for metastasising human melanoma cells in mice (Tasdogan et al., 2020).

Most of the mechanisms described above are designed to supply cancer cell mitochondria with glutamate/α-ketoglutarate to sustain reverse Krebs cycle activity leading to excess citrate synthesis. In this case, glutamate needs to be transported through the inner mitochondrial membrane at a higher rate (Figure 1). Mitochondrial glutamate transporters GC1 (SLC25A22) and GC2 (SLC25A18) are the members of the *SLC25* gene family of mitochondrial carriers. Import of glutamate into mitochondria is dependent on H^+^. Under normal conditions, there exists a H^+^ gradient across the inner mitochondrial membrane in the cytoplasm-to-matrix direction, which provides energy for the activity of GC1 and GC2. High expression of GC1 is associated with organs such as brain, pancreas and the fatty acid synthesizing liver (Monné et al., 2019). GC2 is ubiquitously expressed in most tissues. Another mitochondrial carrier involved in the transport of glutamate into mitochondria is the aspartate-glutamate carrier isoform 1 (AGC1), encoded by *SLC25A12* gene. In this case, glutamate is transported into the mitochondria in exchange for aspartate (Infantino et al., 2019). Because of this exchange phenomenon, the transport function of AGC1 is electroneutral and there is no energy involvement in the process.

Recent studies showed that AGC1 is crucial for different cancer cell types deprived of extracellular glutamine to sustain intracellular aspartate level, cell proliferation and survival (Profilo et al., 2017; Alkan et al., 2018). Intracellular aspartate normally produced in the process of glutamine anaplerosis can be rescued by mitochondrial aspartate synthesis and release through AGC1 into the cytoplasm in case of extracellular glutamine deficiency.

Altogether, based on the published data, it can be concluded that cancer cells engage several different pathways to produce Krebs cycle intermediates. Their import into mitochondria can sustain increased synthesis of citrate using the reductive carboxylation pathway.

Once produced within the mitochondrial matrix, citrate normally enters the Krebs cycle. However, if the cells are energy sufficient, Krebs cycle is suppressed and citrate now is exported into cytoplasm through the mitochondrial citrate carrier mCiC (SLC25A1) in exchange for malate (Iacobazzi et al., 1996). Consistent with the notion of cancer cells producing citrate intracellularly, mCiC expression was found to be increased in different tumor types (Catalina-Rodriguez et al., 2012; Kolukula et al., 2014) and in pathophysiological conditions such as hypoxia. Blocking of the citrate transport from mitochondria into the cytoplasm with an SLC25A1 inhibitor, benzenetricarboxylate (BTA), had several effects on cancer cells including increased glycolysis, ROS synthesis, mitophagy and disrupted mitochondrial homeostasis (Catalina-Rodriguez et al., 2012; Kolukula et al., 2014; Fernandez et al., 2018). Moreover, *in vivo* treatment with BTA resulted in a decreased subcutaneous tumor growth of human breast cancer cell line MDA-MB-231, lung cancer cell line H1299 and bladder cancer line xenografts (Catalina-Rodriguez et al., 2012; Kolukula et al., 2014). Due to its ability to regulate ROS synthesis, mCiC was found to play a major role in acquiring resistance to anti-cancer treatments (Fernandez et al., 2018). Consistently, blocking of SLC25A1 with BTA sensitized the cells to ionizing radiation through increased ROS synthesis. Moreover, citrate carrier expression was found to be increased under hypoxic conditions. Expression of SLC25A1 in patients with non-small cell lung carcinoma has been correlated with decreased survival (Hlouschek et al., 2018).

The results described above highlighting the role of mCiC in the regulation of glycolysis, mitochondrial activity and metabolism under hypoxia contain some controversies. Increased expression of mCiC under hypoxic conditions should not be able to effectively rescue mitochondrial citrate synthesis when no oxygen is present. Moreover, blocking of mCiC with BTA should decrease ROS synthesis due to the decreased mitochondrial activity rather than increase it (Fernandez et al., 2018). Most of these discrepancies could be potentially explained by our recent data showing that cancer cells of different origin can take up extracellular citrate through the plasma membrane citrate transporter pmCiC (Mycielska and Geissler, 2018; Mycielska et al., 2018, 2019), which is a variant of the mitochondrial SLC25A1 (mCiC). pmCiC has a different start codon which is located in the intron preceding second exon of the mCiC (Mazurek et al., 2010). The two variants have different first exons with the remaining sequences identical between the two variants. Therefore, both transporters should be sensitive to the same blockers. Moreover, the primers, siRNAs and antibodies used in these studies will likely not distinguish between the two variants. This would indicate that BTA or siRNAs used are likely to affect both transporters and therefore produce a rather complex effect. Lack of the import of extracellular citrate by cancer cells subjected to such blockers or siRNAs would explain some of the described above results. First, blocking of extracellular citrate uptake could explain increased need of citrate synthesis in mitochondria consistent with increased glycolysis rate and ROS synthesis. In fact, we have shown already that mCiC expression is upregulated in cancer cells in the absence of extracellular citrate and the same holds true for the increased ROS synthesis (Mycielska et al., 2018). Second, increased expression of mCiC alone in hypoxia would not make much sense, as there is little oxygen available thus reducing mitochondrial activity. However, increased citrate import through pmCiC could explain the overall increase in the expression of mCiC/pmCiC as observed in different studies. Increased uptake of extracellular citrate under hypoxic conditions could rescue the overall cancer cell metabolism and sustain fatty acid synthesis. Finally, it will also be important to check the differential expression of mCiC and pmCiC in patient samples and study the correlation between the expression level of each of the variant and the overall survival.

SLC13A5 as another contributor to cytoplasmic citrate detected in cancer cells. SLC13A5 is a Na^+^-coupled citrate transporter (also called NaCT) expressed in the plasma membrane of certain cell types (Inoue et al., 2002a,b, 2004; Gopal et al., 2015). It is expressed predominantly in liver, brain, and testes, with liver being the tissue of the most abundant expression. The location of the transporter in hepatocytes is restricted to the blood-facing sinusoidal membrane, indicating that the physiologic role of this transporter is to deliver citrate from the circulation into the cells (Gopal et al., 2007). Loss-of-function mutations in SLC13A5 cause a severe disease, known as Early Infantile Epileptic Encephalopathy-25 (EIEE-25) (Bhutia et al., 2017; Jaramillo-Martinez et al., 2020), which highlights the biologic role of the transporter that is obligatory for brain function. Surprisingly, *Slc13a5*-null mice are phenotypically normal with no evidence of brain dysfunction (Birkenfeld et al., 2011), underlining potential differences between mice and humans in terms of the involvement of the transporter in brain function. However, the *Slc13a5*-null mice are resistant to diet-induced obesity, insulin resistance and metabolic syndrome (Birkenfeld et al., 2011; Bhutia et al., 2017; Willmes et al., 2018; Jaramillo-Martinez et al., 2020), which is obviously related to the role of the transporter in the liver. We have shown that SLC13A5 delivers extracellular citrate into the liver carcinoma cell line HepG2 for subsequent lipid synthesis (Inoue et al., 2003). Therefore, SLC13A5 ought to play a critical role as a determinant of cytoplasmic citrate at least in the liver, brain and testes. Given that citrate in the cytoplasm is obligatory for fatty acid and cholesterol synthesis and for promotion of cancer cell growth and proliferation, SLC13A5 must have relevance to cancer, at least in the liver where its expression is the most abundant. This is supported by a recent study in which SLC13A5 was shown to be a tumor promoter in hepatocellular carcinoma (Li et al., 2017; Peters, 2017). Silencing the transporter in the liver cancer cells led to decreased ATP, increased AMP-dependent kinase activity, and decreased mTORC1 signaling, consequently resulting in decreased growth of HepG2 cells into tumor in mouse xenografts.

## Citrate Metabolism in Cytoplasm

We have shown recently that cancer cells take up extracellular citrate through the pmCiC (Mycielska et al., 2015, 2018; Mycielska and Geissler, 2018), and others have shown a similar role for the Na^+^-coupled citrate transporter NaCT (SLC13A5) (Inoue et al., 2003; Li et al., 2017; Kopel et al., 2020). Expression of pmCiC was found to correlate with the aggressiveness of cancer in human tissues and to be increased at the invasion front and at the sites of metastasis. *In vitro*, cancer cells incubated with extracellular citrate required less glucose, produced less ROS and decreased their mitochondrial activity (Mycielska et al., 2018). The presence of extracellular citrate reduced expression of mCiC in mitochondria consistent with the reduced release of this metabolite into the cytoplasm and increased uptake of citrate from the extracellular space. Extracellular citrate could directly supply cytosolic pathways and/or be incorporated into the Krebs cycle. However, reduced mCiC expression contradicts the latter possibility. The role of NaCT (SLC13A5) in supplying extracellular citrate has been documented at least in hepatocellular carcinoma and liver cancer cells (Inoue et al., 2003; Li et al., 2017; Kopel et al., 2020); this process is essential for the growth and proliferation of liver cancer cells *in vitro* and *in vivo*.

As in normal cells, cytoplasmic citrate could feed into several metabolic pathways and facilitate overall cancer cell metabolism. Citrate is the primary substrate for fatty acid and cholesterol synthesis; both of these two pathways occur in the cytoplasm. Therefore, citrate entering the cytoplasm via either pmCiC or NaCT could feed into these biosynthetic pathways directly. Citrate in the cytoplasm is metabolized by ATP-citrate lyase to acetyl-CoA and oxaloacetate (Table 1). Acetyl-CoA is then converted into malonyl CoA by acetyl-CoA carboxylase whilst oxaloacetate either enters into gluconeogenesis or gets metabolized to malate (malate dehydrogenase) and transported back to mitochondria through mCiC (Iacobazzi et al., 1996). For the entry of oxaloacetate into gluconeogenesis, the cytoplasmic enzyme phosphoenolpyruvate carboxykinase (PEPCK-C) is needed. It is an GTP-requiring enzyme. Oxaloacetate generated within the mitochondrial matrix via carboxylation of pyruvate by pyruvate carboxylase or via the TCA cycle using the various intermediates in the pathway (α-ketoglutarate, succinyl-CoA, and fumarate) could also enter gluconeogenesis via the mitochondrial PEPCK (PEPCK-M), and the resultant phosphoenolpyruvate is transported out of the mitochondria to feed into the gluconeogenic pathway in the cytoplasm. Interestingly, under glucose deprivation, cancer cells use mitochondrial PEPCK to support cell proliferation. In this case, glutamine is used as the primary substrate to produce phosphoenolpyruvate via oxaloacetate (glutamine → glutamate → α-ketoglutarate → succinyl-CoA → succinate → fumarate → malate → oxaloacetate) in the TCA cycle to supply the carbon skeleton for gluconeogenesis in the cytoplasm (Vincent et al., 2015). Similarly, cytosolic PEPCK has been determined to account for the switch from glutaminolysis to increased glucose consumption in case of glutamine restriction. Under the conditions of limited glucose supply, cancer cells also use glutamine to generate oxaloacetate, which enters the gluconeogenesis pathway in the cytoplasm to synthesize glucose-6-phosphate, which then feeds into the pentose-phosphate pathway to generate NADPH as a means to support the antioxidant machinery (Montal et al., 2015). Acetyl-CoA is used as the building block for the synthesis of fatty acids as well as cholesterol. Similar to fatty acids which are needed for the synthesis of phospholipids necessary for membrane biogenesis, cholesterol also serves an essential function in biomembranes, both being crucial for cancer cells (Coleman, 1986; Rao and Coleman, 1989). Accumulation of cholesterol in the mitochondrial membrane is responsible for its decreased permeability resulting in a tighter regulation of metabolite exchange with the cytoplasm (Baulies et al., 2018). Cholesterol plays a similar role in the control of permeability in the plasma membrane. Cytoplasmic citrate does not function simply as a precursor of acetyl-CoA to serve as the substrate for acetyl-CoA carboxylase; it is also a potent allosteric activator of this enzyme, thus promoting the synthesis of malonyl CoA. Interestingly, malonyl CoA feeds into fatty acid synthesis and at the same time blocks the entry of fatty acids into mitochondria by inhibiting the carnitine palmitoyl transferase 1. As such, malonyl CoA promotes fatty acid synthesis in the cytoplasm and simultaneously blocks breakdown of fatty acids inside the mitochondria. Thus, ATP-citrate lyase is obligatory for most of the anabolic pathways in the cytoplasm involving citrate. As such, this enzyme plays an essential role in cancer cells. Consequently, ATP-citrate lyase has been suggested as an anti-cancer drug target (Hatzivassiliou et al., 2005). Another function of acetyl-CoA in the cytoplasm is in the post-translational modification of enzymes via acetylation, which could impact on their biological function. Acetyl-CoA can also enter the nucleus to modify histones with acetylation, which plays an important role in epigenetic control of transcription (Wellen et al., 2009).

To enhance fatty acid synthesis, cancer cells not only need increased citrate supply in the cytoplasm but also increased supply of NADPH as the reducing equivalent as synthesis of one palmitate requires 14 NADPH molecules (Hochachka et al., 2002). The primary source of NADPH in mammalian cells is the pentose-phosphate pathway (PPP). This pathway is particularly pronounced in cancer cells, supplying pentose phosphates necessary for nucleic acids synthesis and NADPH necessary for anabolic pathways such as the fatty acid/cholesterol synthesis (Patra and Hay, 2014). The PPP consists of two arms: oxidative and non-oxidative. Diversion of PPP into oxidative arm occurs in cells which need to produce NADPH and maintain their redox balance while non-oxidative PPP is used to support nucleic acids synthesis and further proliferation. To support the increased demand for ribose-5-phosphate, cancer cells increase the non-oxidative arm of the PPP by regulating expression of specific target enzymes in the pathway (Langbein et al., 2006; Liu et al., 2010). Phosphoglycerate mutase (PGAM) is a dimeric enzyme comprising of two subunits PGAM1 and PGAM2. The tumor suppressor p53 elicits a negative effect on the oxidative arm of PPP by suppressing the glycolytic enzyme PGAM1 (phosphoglycerate mutase 1). As p53 gets mutated in a majority of cancers, this effect is reversed in cancer cells resulting in increased expression and activity of PGAM1. Since PGAM1 converts 3-phosphoglycerate into 2-phosphoglycerate in glycolysis, an increase in the activity of this enzyme in cancer cells causes a decrease in 3-phosphoglycerate. 6-Phosphogluconate dehydrogenase is an enzyme in the oxidative arm of PPP and this enzyme is inhibited by 3-phosphoglycerate. As such, the decrease in 3-phosphoglycerate in cancer cells relieves this inhibition and consequently potentiate the oxidative arm of PPP, thus resulting in increased generation of NADPH (Hitosugi et al., 2012).

NADPH can also be synthesized as a by-product in another cytosolic pathway involving citrate metabolism via cytosolic aconitase (c-ACN). c-ACN is an interesting protein known more commonly as Iron Regulatory Protein1 (IRP1). When intracellular Fe is low, IRP1 upregulates expression of proteins responsible for Fe uptake. In the presence of increased levels of intracellular Fe, IRP1 forms Fe-S clusters which prevent its binding to RNA; thus the protein loses its function as IRP1 but acquires activity of c-ACN (Bodiga and Krishnapillai, 2007; Wang et al., 2008). c-ACN metabolizes citrate into isocitrate which is then transformed into α-ketoglutarate with a concomitant synthesis of NADPH, the latter step mediated by cytosolic isocitrate dehydrogenases 1 and 2 (IDH1/2), which generate NADPH in contrast to the mitochondrial IDH (i.e., IDH3) which generates NADH. α-ketoglutarate is used in the Krebs cycle. Consistently, inhibition of c-ACN resulted in decreased NADPH:NADP ratio and decreased expression of genes associated with lipogenesis during differentiation of human adipocytes (Moreno et al., 2015). This would strongly suggest that increased synthesis of fatty acids requires activity of c-ACN.

The cytosolic c-ACN functioning as IRP1 as well as the enzyme in the conversion of citrate into isocitrate enables a cross-talk between cellular iron status and fatty acid synthesis. Cancer cells reprogram iron-regulatory pathways to increase iron uptake (Jung et al., 2019). Fe in blood is complexed with transferrin (Tf) and taken up by cells through TfR1 (Tf receptor 1). TfR1 expression has been found not only to be increased in cancer cells but also to correlate with increased motility, proliferation and adhesion, and also increased resistance to chemotherapy (Greene et al., 2017). Moreover, cancer cells express increased levels of DMT1 (divalent metal transporter 1; Xue et al., 2016) in endosomes facilitating Fe transport into the cytoplasm following TfR1-mediated endocytosis of transferrin-bound iron. Cancer cells also suppress the expression of the iron exporter ferroportin (SLC40A1), thus enhancing iron retention inside the cells (Kong et al., 2019). The expression of ferritin, responsible for iron storage inside the cells, is also expressed at higher levels in cancer cells (Wang et al., 2018). Increased intracellular Fe levels in cancer cells (Brown et al., 2020a) will facilitate the transition of c-ACN from IRP1 to the mediator of citrate-to-isocitrate conversion. With pmCiC and NaCT functioning to bring extracellular citrate into the cancer cells, excess iron promotes the conversion of citrate into isocitrate in the cytoplasm via c-ACN with subsequent generation of NADPH. Therefore, this pathway could provide another way of supplying cancer cell cytoplasm with NADPH and help keeping redox balance. In this way, influx of extracellular citrate could release not only mitochondria from excess citrate synthesis, reduce ROS production (therefore increase resistance to chemotherapy) but also allow the use of other metabolic pathways like PPP in the most beneficial way for cancer cells. It is therefore not surprising that chronic exposure to excess iron as occurs in the genetic iron-overload disease hemochromatosis transforms normal cells into cells with a tumor phenotype (Gnanaprakasam et al., 2013; Bhutia et al., 2020) and also increases the risk of cancer, particularly liver cancer (Grosse et al., 2018) and colon cancer (Sivaprakasam et al., 2020).

## Metabolic Crosstalk Between Cancer and Its Environment

**Figure 2:**
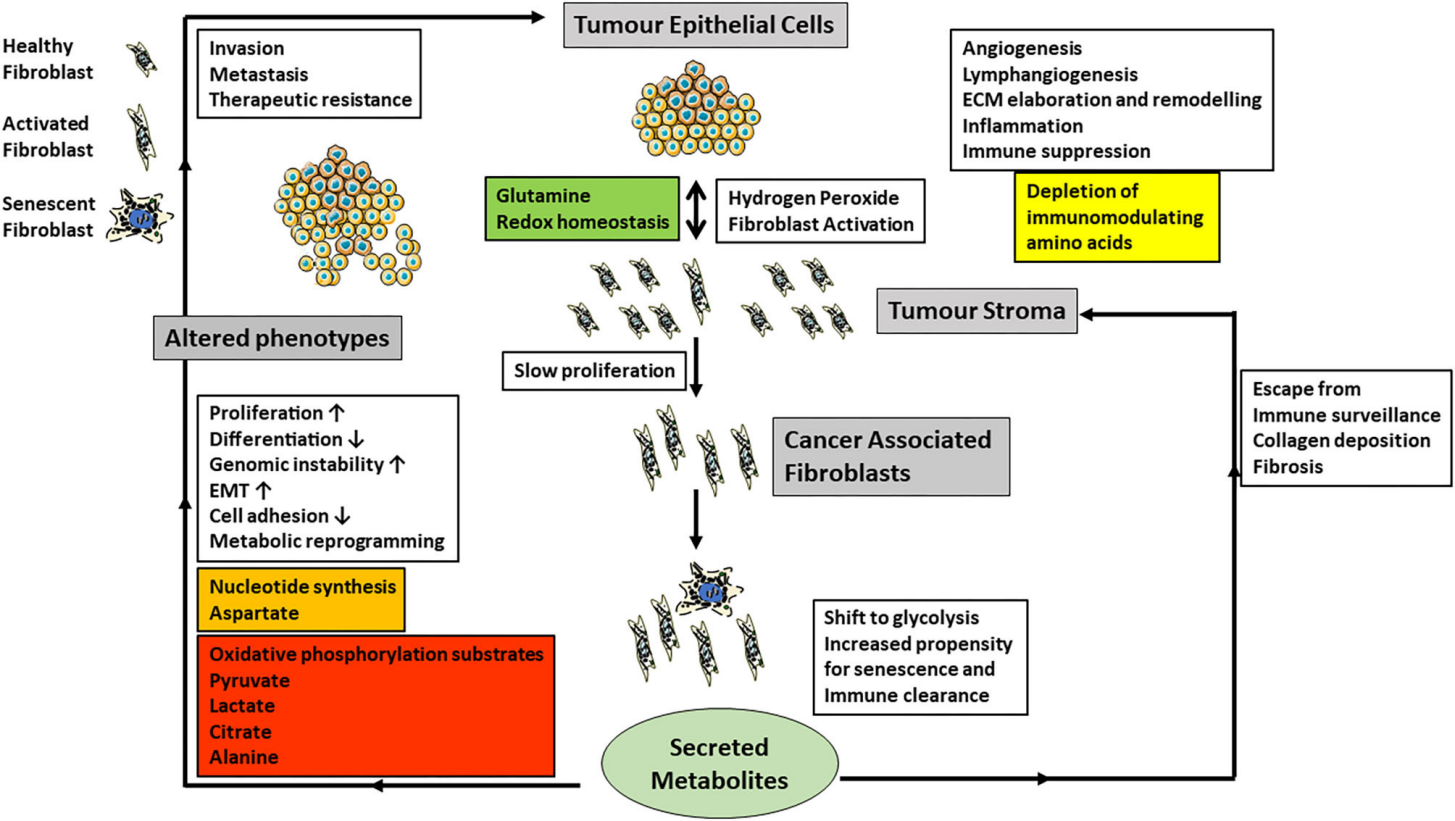
The cartoon summarizes current data on the role of CAF-derived metabolites and the regulation of cancer phenotypes (gray boxes) and especially the influence CAFs have on epithelial-mesenchymal transition (EMT), invasion, metastasis and angiogenesis (white boxes). Carcinoma cells produce excess hydrogen peroxide and induce fibroblast activation in neighboring fibroblasts, which also have an increased propensity to undergo senescence. Senescent cells are normally cleared by the immune system. However, if they escape, they also produce tumor promoting stimuli, which are similar but not the same as those of CAFs. Metabolically CAFs and senescent fibroblasts modulate redox homeostasis and shift their metabolism toward glycolysis and the extracellular metabolites produced are summarized in the colored boxes. In particular, the secretion of energy metabolites to drive carcinoma cell energy and fatty acid metabolism as well as epigenetic regulation may be particularly important.

Extracellular metabolic support is crucial for cancer cells, and cancer-associated stroma can potentially supply necessary metabolites. In recent years it has been realized that cancer operates as a dysfunctional and constantly evolving tissue rather than merely clonal evolution with the neoplastic cells themselves (Hanahan and Weinberg, 2000). The role and composition of the cancer microenvironment has been extensively reviewed in recent years (Sahai et al., 2020) and will only be summarized briefly here (Figure 2). The tumor microenvironment is composed of a distinctive microbiome, blood vessels, cells of the adaptive and innate immune systems and stromal cells which include cancer-associated fibroblasts (CAFs), which have been proposed as a novel cellular target for cancer therapy. The origin, defining features and properties of CAFs have been reviewed recently (Sahai et al., 2020). CAFs secrete a number of molecules that include transforming growth factor beta, vascular endothelial growth factor, platelet-derived growth factor, collagens, matrix metalloproteinases and cytokines. CAFs are also prone to undergo senescence albeit at a low frequency and exhibit a reduced replicative lifespan *in vitro* (Pitiyage et al., 2011; Costea et al., 2013; Hassona et al., 2013). The properties of CAFs and senescent fibroblasts overlap substantially (Lim et al., 2011; Faget et al., 2019; Sahai et al., 2020) but are not identical (Mellone et al., 2016). Fibroblast populations from pre-malignant (Pitiyage et al., 2011) and malignant (Mellone et al., 2016) lesions are composed of pro-fibrogenic and anti-fibrogenic cells.

The exchange of metabolites between cancer cells and CAFs has only been investigated relatively recently and has produced a variety of observed relationships between CAFs and various types of cancer (Martinez-Outschoorn et al., 2014; Valencia et al., 2014; Bertero et al., 2019; Sanford-Crane et al., 2019; Sahai et al., 2020). However, one of the central observations is that CAFs shift their energy metabolism toward glycolysis (Kalluri, 2016) and advanced senescent fibroblasts likewise (James et al., 2015, 2018). In addition, senescent cells upregulate the PPP (James et al., 2015), NADPH (James et al., 2016), several lipids (James et al., 2016) and PGAM2 transcript (James et al., 2015) and so in these respects they resemble cancer cells but possess low chronic wild type p53 activity (Wiley and Campisi, 2016). The activation of fibroblasts into CAFs also promotes catabolism and autophagy (Kalluri, 2016). In prostate (Ippolito et al., 2019) and squamous cell carcinoma (Zhang et al., 2020), CAFs promote oxidative phosphorylation, epithelial-mesenchymal transition (Ippolito et al., 2019), tumorigenicity (Zhang et al., 2020), increased metastatic spread and drug resistance by secreting lactate (Ippolito et al., 2019; Zhang et al., 2020). There is a reciprocal relationship between CAFs and squamous cancer cells where glutamine provided by the cancer cells maintains redox homeostasis in the CAFs. The CAFs in turn release aspartate to promote nucleotide synthesis and a reduction in both amino acids in tumors reduces proliferation (Bertero et al., 2019). Pyruvate release by CAFs promotes lymphoma cell survival by upregulating the Krebs cycle (Sakamoto et al., 2019) and promotes extracellular matrix remodeling and breast cancer metastasis via α-ketoglutarate (Elia et al., 2019). Lactate is another tumor cell-derived metabolite that elicits a pivotal cross-talk with the tumor microenvironment. Extracellular lactate, generated and then released by cancer cells, acts as a signaling molecule via its receptor GPR81 expressed on immune cells present in the tumor microenvironment; this cross-talk facilitates immune evasion of the tumor cells (Brown et al., 2020). Lactate is not the only signaling molecule for the cross-talk between tumor cells and stromal cells. The ketone body β-hydroxybutryate also functions as a signaling molecule to facilitate communication between cancer cells and stromal cells (Ristic et al., 2017). Autophagy in stromal fibroblasts which is associated with both CAFs and senescence can generate alanine to fuel the Krebs cycle of pancreatic adenocarcinoma cells (Sousa et al., 2016; Sanford-Crane et al., 2019). Metabolic dysregulation of CAFs may also be coupled to altered immunoregulation, possibly through IL-6 production or depletion of immunomodulating amino acids (Valencia et al., 2014). Although many metabolites have now been implicated in the crosstalk between cancer cells and their environment, none of these studies as yet has focused on citrate which lies at the crossroads of energy and lipid metabolism as well as epigenetic regulation. With regard to citrate in the tumor microenvironment, two aspects seem very relevant. First, the acidic pH in the tumor microenvironment is important for the entry of extracellular citrate into cancer cells via pmCiC and NaCT. As citrate is a trivalent anion under physiological pH, its presence in the single-protonated divalent anionic form for transport via pmCiC is facilitated by the acidic tumor microenvironment. Similarly, even though NaCT is a Na^+^-coupled transporter for citrate, its transport function is markedly stimulated by extracellular acidic pH because one of the Na^+^-binding sites shows much higher affinity for H^+^ than for Na^+^ (Gopal et al., 2015). Second, citrate in the tumor microenvironment as a promoter of tumor cell growth becomes especially important metastatic tumors growing within the bone. Citrate is present at high concentrations in the bone matrix; in fact, ~70% of citrate in the body resides within the bone. When metastatic tumors grow within the bone matrix, tumor cells secrete metalloproteinases to degrade bone matrix, thus essentially dissolving the bone mineral. This process releases citrate in the microenvironment. As such, tumor cells growing in bone are exposed to high levels of citrate in the microenvironment, which is available to support the tumor cell growth.

## Conclusions

In the process of metastasis and distant organ colonization, cancer cells are faced with changing extracellular conditions including availability of different metabolites. Based on the available literature, we have shown here that cancer cells have a remarkable flexibility in using different pathways to overcome shortage in certain metabolites. This makes their metabolism adjustable and sustainable regardless of the extracellular conditions. Citrate supply and synthesis, discussed in this review, can be used as an example of how cancer cells account for the missing metabolites. In this case, several mechanisms are employed including serine/glycine pathway, truncated and reverse Krebs cycle or lactate uptake to boost mitochondrial citrate synthesis. Most of these reprogrammed metabolic pathways have been shown to function *in vitro*. It is however possible that *in vivo* where the surrounding conditions are less friendly, cancer cells must find a more stable way of getting the supply of the necessary metabolites, which could come from the stromal cells. It is important to fully understand the metabolic addictions of cancer cells and their reliance on the cancer-associated stroma to be able to devise novel and more efficient and selective strategies to combat cancer.

## Author Contributions

All authors listed have made a substantial, direct and intellectual contribution to the work, and approved it for publication.

## Conflict of Interest

The authors declare that the research was conducted in the absence of any commercial or financial relationships that could be construed as a potential conflict of interest.
